# Idiopathic Pleuroparenchymal Fibroelastosis

**DOI:** 10.1007/s13665-017-0160-5

**Published:** 2017-01-27

**Authors:** Martina Bonifazi, M. Angeles Montero, Elisabetta A. Renzoni

**Affiliations:** 1 Department of Biomedical Sciences and Public Health, Università Politecnica delle Marche, Ancona, Italy; 2 Pulmonary Diseases Unit, Department of Internal Medicine, Azienda Ospedali Riuniti, Ancona, Italy; 3 Department of Histopathology, Imperial College, London Royal Brompton and Harefield Hospitals NHS Foundation Trust, London, UK; 4 Interstitial Lung Disease Unit, Royal Brompton Hospital, Imperial College, London, UK

**Keywords:** Pleuroparenchymal fibroelastosis, Idiopathic interstitial lung disease, Review, Diagnosis, Clinical features

## Abstract

**Purpose of the review:**

Idiopathic pleuroparenchymal fibroelastosis (IPPFE) is a rare fibrosing lung disease, affecting the visceral pleura and the subpleural parenchyma with an upper lobe predilection, included as a distinct clinicopathologic entity in the latest international multidisciplinary classification of the idiopathic interstitial pneumonias (IIP). We aim to summarize the current evidence on IPPFE, in terms of clinical features and potential treatments.

**Recent findings:**

Overall, there is increasing awareness of PPFE in association with a separate ILD pattern. Although an agreed consensus on diagnosis has yet to be defined, a list of radiological and histopathological criteria has been proposed. Due to the unfavorable risk-benefit profile of surgical lung biopsy in a significant proportion of patients, a potential role for transbronchial lung cryobiopsy has been suggested. At present, lung transplantation remains the only curative option.

**Summary:**

The increasing awareness of this condition among specialists has led to more frequent identification of IPPFE. Large international studies are needed to better characterize pathogenesis and pheno/endotypes of disease, a key step towards the development of effective treatments.

## Introduction

Pleuroparenchymal fibroelastosis (PPFE) is a rare interstitial lung disease, characterized by dense fibrosis of the visceral pleura and the subjacent lung parenchyma, with a striking upper lobe predominance. Although sporadic cases of upper lobe dominant fibrosis of unknown origin had been previously published [1], Amitani et al. first described this peculiar condition in a series of patients in 1992 [2]. Since then, a number of similar cases have been reported in the Japanese literature under the term of “Amitani’s disease” or idiopathic “pulmonary upper-lobe fibrosis” (PULF). The currently accepted term PPFE first appeared in the English literature later, in 2004, when Frankel et al. reported five cases of an “idiopathic pleuroparenchymal fibroelastotic syndrome with unique pathology” that could not be classified into any of the previously recognized interstitial pneumonias. Although the acronym refers to a specific morphological pattern, PPFE is now widely considered as a distinct clinicopathological entity, with definite radiological and pathological characteristics, and has been included in the latest international multidisciplinary classification of the idiopathic interstitial pneumonias (IIP), in the rare entities section [3]. To date, little more than one hundred cases have been reported overall in the published literature, but increasing awareness of this entity among specialists will likely lead to more precise epidemiological estimates on its actual occurrence.

The etiology has yet to be clearly established, but a large proportion of reported PPFE cases has been described in association with lung, bone marrow, and hematopoietic cell transplantations [4], in association with a number of chemotherapy drugs [5, 6], as well as with occupational exposures such as aluminosilicate dust [7]. In the case series by Reddy et al. [8••], approximately half of cases reported a history of recurrent lower respiratory tract infections, suggesting that repeated inflammatory damage caused by pulmonary infections may contribute to the development/progression of PPFE in a proportion of patients. PPFE has also been reported in association with a variety of separate ILD patterns, including idiopathic pulmonary fibrosis (IPF) [8••, 9•, 10]. Moreover, it can occur in the context of familial forms of IIP, mostly in young females [11, 12], suggesting the potential role of a genetic predisposition. When none of the associated conditions are identified, PPFE is considered idiopathic (IPPFE). In keeping with the scope of the present review, we will mainly focus on IPPFE, summarizing current evidence and latest data on clinical features, radiological characteristics, histological findings, differential diagnosis, prognosis, and potential treatments.

## The Clinical Spectrum

IPPFE usually presents in adults, but the age at onset is highly heterogeneous, ranging from 13 to 87 years, with a median value of approximately 53 years [13]. In particular, a bimodal distribution of age at presentation has been observed, with an earlier peak in the third decade, and a later one in the sixth decade. A part from a female predominance in younger patients, the overall male to female ratio does not reveal a significant gender predilection [14•]. Cigarette smoking does not appear to be a risk factor. Indeed, PPFE mostly occurs in non-smokers [15].

The main clinicopathologic features are summarized in Table 1. Clinical features include exertional dyspnoea, cough, weight loss, and chronic dull pleuritic pain, which may intensify over time, even in absence of an underlying pneumothorax. Episodes of uni- or bilateral pneumothoraces occur in up to one third of patients. Moreover, the spontaneous co-occurrence of pneumothorax and pneumoperitoneum has been recently described [16]. Pneumothoraces in these subjects rarely resolve spontaneously, and persistent air leaks are often observed

**Table 1:** Main clinicopathologic features of idiopathic pleuroparenchymal fibroelastosis (IPPFE)

Demographic aspects
Variable age at onset (range 13–87, median value 53 years)
No gender predilection
Clinical history
Mostly non-smokers
Recurrent pulmonary infections
Familial background of interstitial lung disease
Symptoms
Dry cough
Exertional dyspnea
Chronic dull pleuritic pain (occasionally sharp)
Weight loss
Signs
“Flattened” thoracic cage (or “plathythorax”)
Slender habitus
Bibasal crackles, if usual interstitial pneumonia (UIP)-like changes in lower lobes
Functional parameters
Restrictive ventilatory impairment: disproportionate reduction in forced vital capacity (FVC) compared to diffusing capacity of carbon monoxide (DLCO), with KCO (DLCO/VA) trends towards supernormal values
Increased ratio of residual volume/total lung capacity (RV/TLC)
Gas analysis
Earlier stages: normal pressure of oxygen (Pa O2), with mild increase in the partial pressure of carbon dioxide (PCO2), with a preserved alveolar-arterial gradient of oxygen (A-aDO2)
Advanced stages: hypoxemia with hypercapnic respiratory failure
Serum biomarkers
Elevated surfactant protein D (SP-D)
Normal Krebs von den Lungen-6 (KL-6), or slightly increased in advanced stage
Increased titres of a variety of serum auto-antibodies
Imaging features
Upper lobe bilateral, irregular pleural thickening, and dense reticular fibrosis of subjacent lung parenchyma
Clear demarcation between abnormal and normal lung
Hila retracted upwards with distortion of the lung architecture
Overall volume loss and reduced ratio of the anteroposterior to the transverse diameter
Interlobular septal thickening, small foci of consolidation, large cysts, and multiple bullae may be observed
Uni- or bilateral pneumothoraces may occur
Coexistent interstitial involvement of the lower lobes (usually UIP-like changes) in a proportion of cases
Pathologic findings
Fibrous thickening of the visceral pleura with elastic fibers
Homogeneous, dense, intra-alveolar fibrosis with septal elastosis (twice that observed in idiopathic pulmonary fibrosis)
Transition from abnormal lesions to normal tissue typically abrupt
Mild, sparse mononuclear lymphocytic infiltration
Sparse fibroblastic foci
Partial stenosis of pulmonary vessels, both arterial and venous

.

Patients are often slender and may present with plathythorax, especially in advanced stages. Such a deformity of the thoracic cage, measured as a reduced ratio of the anteroposterior to the transverse diameter at computed tomography (CT), has been defined as “flat chest” by the authors who first identified this peculiar physical finding. The flattened thoracic cage may worsen during the disease course along with the functional decline, suggesting that an acquired defect due to the decreasing volume of the upper lobes is a more likely explanation than a congenital predisposition [17]. On physical examination, inspiratory crackles are rare and may be the expression of concomitant usual interstitial pneumonia (UIP)- or non-specific interstitial pneumonia (NSIP)-like changes in the lower lobes. Digital clubbing is usually absent.

Lung function tests are usually characterized by a restrictive ventilatory impairment, with markedly decreased forced vital capacity (FVC) and slightly reduced total lung capacity (TLC). The ratio of forced expiratory volume in one second/FVC (FEV1/FVC) is increased, as is the ratio of residual volume/TLC (RV/TLC), possibly resulting from the compensatory hyperinflation of lower lobes due to the fibrotic collapse of the upper sections of lungs. Watanabe et al. recently investigated the potential coexistence of small airways disease, usually characterized by an increased RV/TLC ratio, in a series of nine patients with IPPFE, but did not identify any radiological signs of air trapping, pathological evidence of bronchiolitis, or functional response to bronchodilators [18]. The diffusing capacity of carbon monoxide (DLco) is overall decreased, with preserved KCO (DLCO/VA), as observed in restrictive impairments [2]. Indeed, because of an element of extrapulmonary restriction caused both by the pleural fibrosis and the thoracic cage abnormalities described above, there may be a disproportionate reduction in FVC compared to DLCO, with the resulting KCO edging towards supernormal values (A/N).

In the earlier stages of the disease, arterial blood gas analysis usually documents a normal partial pressure of oxygen (Pa O2) and a trend towards a mild increase in the partial pressure of carbon dioxide (PCo2) with a preserved alveolar-arterial gradient of oxygen (A-aDO2) [18]. Oxygen desaturation on a six minute walk test (6MWT) is rare. This particular blood gas profile is different from that observed in the other interstitial lung diseases, characterized by decreased values of both PaO2 and PCo2 and early desaturation on exertion. The tendency towards hypercapnia with a normal A-aDO2 gradient in the early stages is again likely related to the significant component of extrapulmonary restriction. With the progression of the fibrotic process, hypoxemia with hypercapnic respiratory failure occurs in the advanced stages.

Elevated serum levels of surfactant protein D (SP-D) may be observed, and Krebs von den Lungen-6 (KL-6) seems to increase as the disease progresses, possibly as expression of alveolar type II cell injury [19]. Increased titres of a variety of serum auto-antibodies may also be detected in a minority of cases, possibly reflecting a pathogenetic role of immune dysregulation [8••, 15].

### Imaging Features

**Fig. 1:**
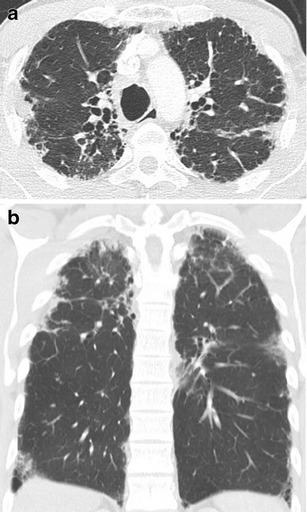
**a** (Trasversal section) pleuroparenchymal irregularity with peaks of fibrosis around the pleural surfaces consistent with PPFE. The proximal airways are abnormally dilated (bronchiectatic), possibly the consequence of repeated infections. **b** (Sagittal section) there is pleuroparenchymal fibrosis with a clear mid and upper zone distribution. In the costophrenic angles, there is evidence of interstitial disease (limited honeycombing) with no conspicuous pleural disease

Chest high resolution CT (HRCT) findings are highly suggestive and include bilateral, irregular pleural thickening and dense reticular fibrosis of subjacent lung parenchyma with upper lobe predominance. The presence of a clear demarcation between the abnormal and normal lung is a characteristic feature (Fig. 1). Wedge-shaped pleural densities advance along parenchymal septa and tend to retract the hila upwards with distortion of the lung architecture. Further associated features are interlobular septal thickening, small foci of consolidation, and volume loss. Moreover, large cysts and multiple bullae may be observed, possibly associated with the high occurrence of pneumothoraces in these patients, along with an altered resistance of the pleura to mechanical stress. Dilated proximal airways, with free standing bronchiectasis (Fig. 1a), are frequently observed, possibly as a consequence of recurrent infectious episodes [20]. The free standing airway dilatation should not be confused with the traction bronchiectasis seen on a background of interstitial fibrosis. As reported above, reduced diameters of the thoracic cage are frequently described.

A coexistent interstitial involvement of the lower lobes away from the pleuroparenchymal fibroelastotic changes is described with increasing frequency in the literature, with the ILD pattern usually reminiscent of UIP- or NSIP-like changes (Fig. 1b) [9•, 10]. Extension of a PPFE pattern to the lower lobes has been demonstrated in a subgroup of patients [21]. These data have been recently confirmed in a relatively large series of Japanese patients (*n* = 21), reporting an equal involvement of upper and lower lobes in approximately one third of cases [22].

### Histological Pattern

**Fig. 2:**
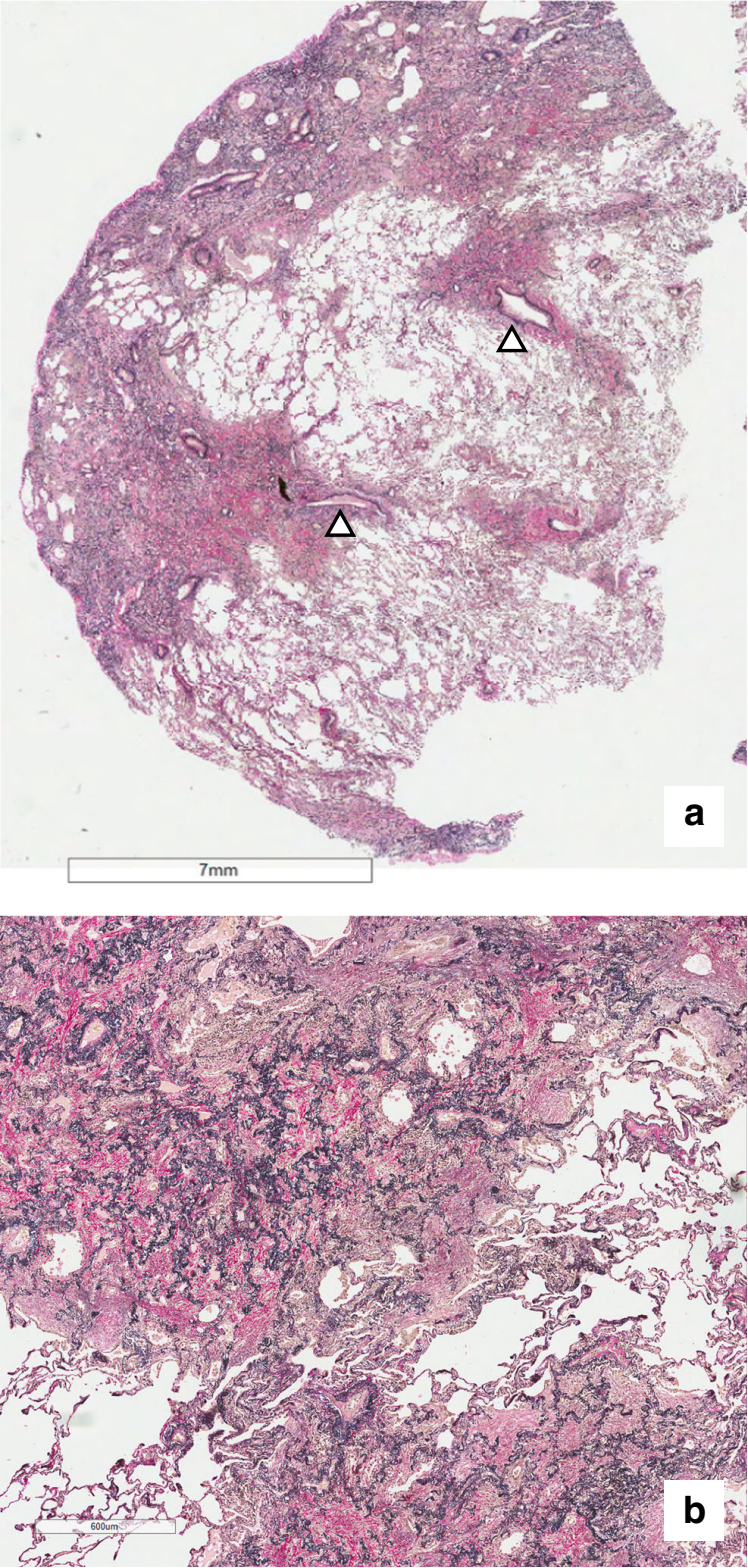
**a** Section of a lung biopsy which displays subpleural and centrilobular fibroelastosis. The vessels show mild fibrointimal thickening (*white arrow*). **b** At higher magnification, the distinctive pattern of PPFE comprises intraalveolar fibrosis and interstitial elastosis (*IAFE*). At the edge, between the IAFE and the lung parenchyma, fibroblast foci are identified (*black arrow*)

The main histological features are fibrous thickening of the visceral pleura and homogeneous, dense intra-alveolar fibrosis with septal elastosis, sharply separated from the adjacent “spared” lung parenchyma. The transition from abnormal lesions to normal tissue is, indeed, typically abrupt. A mild, sparse mononuclear lymphocytic infiltration may be also described, as well as partial stenosis of pulmonary vessels, both arterial and venous. Other upper lobe changes include foci of intra-alveolar fibrosis with septal elastosis with perilobular or bronchocentric distribution. Elastic fibers (EF) within fibrosis are easily identified by using specific stains, such as orcein or Verhoeff van Gieson stains (Fig. 2a, b). Although experienced pathologists may be able to recognize the characteristic features of PPFE on a simple haemotoxylin and eosin stained slide, routine staining for EF in the case of predominantly pleural and subpleural lesions is highly recommended to aid in differential diagnosis. The main mimic in this context is a UIP pattern, be it idiopathic or secondary. Areas of fibroblastic proliferation can be observed in some cases, but the limited number of foci usually permit to distinguish between the two morphological entities [14•]. Moreover, although deposition of elastin is described in UIP, a recent quantitative assessment showed that the amount of EF in IPPFE upper lobes was twice than that observed in IPF and remained significantly higher even when the comparison was made with IPPFE lower lobes [23]. Further relevant differences include the temporal heterogeneity of the fibrosing process and the striking remodeling of lung parenchyma, with architectural disruption. It is worth underlining, however, that these two patterns are not mutually exclusive, as recent studies have revealed that their coexistence may be more frequent than previously thought [9•, 20]. Elements of diffuse alveolar damage, alveolar hemorrhage, and obliterative bronchiolitis can be observed, although this seems to occur in transplant patients, rather than in IPPFE [24].

### Diagnosis

Currently, an agreed consensus statement on IPFFE diagnosis has yet to be defined, although a list of radiological and histopathological criteria have been proposed and widely adopted in the published literature. In 2012, Reddy et al. [8••] suggested both radiological and morphological criteria for “definite,” “consistent with,” and “inconsistent with” PPFE. With reference to imaging, “definite” PPFE referred to cases with pleural thickening and subpleural fibrosis confined, almost exclusively, to the upper lobes; “consistent with” did not necessarily require an upper lobe predominance and allowed features of coexistent disease elsewhere; “inconsistent with,” when the above described features were absent. Histological criteria for “definite” PPFE included upper zone pleural fibrosis with subjacent intra-alveolar fibrosis accompanied by alveolar septal elastosis; a “consistent with” pattern was assigned when intra-alveolar fibrosis was present but it was not associated with pleural fibrosis, was not predominantly beneath the pleura, or was not in an upper lobe biopsy; “inconsistent with,” when the required features described above were absent. Further histological criteria for PPFE diagnosis were proposed by Rosembaum et al. in 2015 [21] as follows: 1) fibrous interstitial pneumonia with 80% fibroelastic changes in nonatelectatic (collapsed) lung; 2) subpleural and/or centrilobular distribution; 3) overall inflammation absent to mild; 4) no specific lobe predilection, typically multilobar; 5) Rare or no granulomas.

While a definite diagnosis of IPPFE would ideally require a combination of radiological and morphological features, in a substantial number of cases, a surgical lung biopsy is not obtained, due to the unfavorable risk-effectiveness profile. IPPFE patients often present with advanced disease with limited ventilatory reserve, and, because of the absence of a curative treatment, the risks related to the surgical procedure, including potentially prolonged post-operative iatrogenic pneumothorax, can be perceived to overcome the potential benefits. On the other hand, particularly when features of an associated ILD pattern without clear-cut diagnostic features are present away from the PPFE changes, a surgical lung biopsy can offer the advantage of obtaining samples from different lobes. More recently, transbronchial lung cryobiopsy (TBLC) has been proposed as a valuable, less invasive, sampling technique in the diagnostic work-up of ILDs [25]. Although the pooled proportion of post-procedural pneumothorax onset from meta-analyses of literature is not negligible (up to 12%) [25, 26] and its frequency may well be higher in the context of PPFE, the risk-effectiveness profile may be reasonable in selected, healthier IPPFE patients. However, as the clinical and radiological features in these patients are often highly suggestive, the need for a morphological assessment has been debated [24] and a label of “consistent with PPFE” for cases without biopsy has been proposed. At any rate, it is unquestionable that a multidisciplinary approach is mandatory for a conclusive diagnosis of IPPFE.

A list of differential diagnoses should be considered while evaluating cases with suspected IPPFE. Asbestos exposure, a previous history of tuberculosis, as well as signs and symptoms suggestive of sarcoidosis and connective tissue disorders should be carefully investigated. Appropriate laboratory tests for autoimmune screening and bronchoalveolar lavage (BAL) analysis may be required in selected contexts. The apical cap is an idiopathic lesion of lung apices, in the form of a subpleural pyramidal scar, histologically characterized by subpleural fibrosis and curls of EF. It can be differentiated from IPPFE, as it does not involve the pleura circumferentially, and it occurs mostly in older males with a history of smoking and tends not to progress over time [14•].

#### Association with other ILD Patterns

A substantial proportion of patients with IPPFE presents with UIP-like features in the lower lobes [10]. Nakatani et al. retrospectively reviewed 205 patients with ILD undergoing lung biopsy and identified 12 PPFE cases, of which 11 had interstitial lung disease in the lower lobes (five definite UIP, four possible UIP, one NSIP, one unclassifiable) [10]. Oda et al. conducted a retrospective review of patients with a histological UIP pattern, diagnosed with IPF, in order to identify the proportion of subjects meeting the above mentioned radiological and morphological criteria for PPFE and to assess whether they presented with distinctive characteristics. Nine of 110 patients (8.2%) met both radiological and histological criteria for IPPFE diagnosis (PPFE/UIP). When compared to the remaining 99 “pure” IPF subjects (IPF/UIP), patients with PPFE/UIP showed flattened chest, a lower BMI, a higher complication rate of pneumothorax and pneumomediastinum, and distinctive functional features, including a preserved DLCO, an increased RV/TLC ratio, and a higher PCO2. Moreover, although not significantly different, the median survival time tended to be shorter. The presence of PPFE was identified in a substantial proportion (33.6%) of large group (*N* = 274) of consecutive patients with a diagnosis of IPF at a single Institution (Royal Brompton Hospital). In this study, the presence of PPFE was associated with a significantly more rapid functional decline and worse survival, even after adjustment for background ILD severity, as assessed by CT extent [20]. Whether PPFE in association with a background UIP pattern should be routinely considered as a poor prognostic marker of IPF, and/or as separate entity requiring target treatment, remains to be established.

### Associated Conditions

It is well known that a PPFE pattern may be also associated with a large variety of conditions, although a clear causative relationship has yet to be established. So far, the strongest association seems to be with a previous organ transplant. In a recent retrospective study, PPFE was detected as a late complication in 7.5% of lung transplantation recipients and in 0.2% of hematopoietic stem cell transplantation recipients [4]. Concurrent histologic features in these patients included acute and/or organizing diffuse alveolar damage, characterized by the presence of intra-alveolar fibrinous exudates appearing to merge into PPFE lesions and constrictive bronchiolitis. PPFE may, thus, represent the final expression of a multifactorial intra-alveolar lung injury, as a number of possible causes may contribute to its onset, including previous chemotherapy regimens, radiation, recurrent infections, and graft versus host disease. Previous exposure to alkylating agents, such as cyclophosphamide and carmustine, has been described as potentially associated with PPFE, although the time between the last drug intake and the first noticeable symptom ranged from 1 to 16 years. As mentioned above, a concomitant role of recurrent infections cannot be ruled out, with a proportion of patients reporting recurrent infections during the course of disease [8••]. In particular, Aspergillus and Mycobacterium avium intracellulare infections have been reported [15, 27].

### Prognosis and Management

Prognosis has been reported as highly variable and largely unpredictable. So far, limited data are available on IPPFE evolution, as, in most of the studies, the overall survival rate is reported together for idiopathic and secondary forms. Overall, the clinical course in idiopathic cases seems to be rapidly progressive, especially in young females with a familial background and in the subgroup of patients with lower lobe UIP features [9•, 20]. However, there are cases of IPPFE which remain relatively stable in the longer term. Due to the long subclinical phase, IPPFE is commonly diagnosed in the advanced stages, making a reliable assessment of disease behavior since its first onset difficult [2]. Yoshida et al. have recently identified two distinct patterns of lung function decline in their cohort of patients, a rapid decline in FVC over a short period, and a slow decline over a longer period, although the latter was observed only in one quarter of subjects [22].

At present, management is challenging, as no treatment has yet been shown to modify the natural course of disease. Patients have been empirically treated with corticosteroids, immunosuppressive agents, and N-acetyl cysteine, with transient or no benefit [28]. A potential efficacy of pirfenidone in preventing lung function decline has been suggested in a recent case-report [28]. An interesting temporal relationship was observed in this patient between lung function decline and pirfenidone intake, as the disease stabilized while taking the drug, progressed after drug discontinuation due to liver toxicity, and then stabilized again once pirfenidone was reintroduced. Supportive care includes oxygen therapy in case of respiratory failure and careful infection control. In advanced stages, lung transplantation should be considered, as cases of successful lung transplants in IPPFE patients have been described [15].

## Conclusions

IPPFE is a rare condition, characterized by distinctive clinical, radiologic, and pathologic features that warrant its inclusion as a separate entity in the latest classification of IIP. Although IPPFE pathogenesis remains largely unclear, the heterogeneous spectrum of clinical presentation and behavior suggests that it may represent the final expression of a variable interplay between environmental exposure, immune dysregulation, and genetic predisposition. The increasing awareness of this condition among specialists has led to the more frequent identification of IPPFE, revealing that it may not be as rare as previously perceived. Large international studies are urgently needed to better characterize the different pheno/endotypes of disease and to develop effective treatments.

## References

[1] Davies D, Crowther JS, MacFarlane A (1975). Idiopathic progressive pulmonary fibrosis. Thorax.

[2] Watanabe K (2013). Pleuroparenchymal fibroelastosis: its clinical characteristics. Curr Respir Med Rev.

[3] Travis WD, Costabel U, Hansell DM (2013). An official American Thoracic Society/European Respiratory Society statement: update of the international multidisciplinary classification of the idiopathic interstitial pneumonias. Am J Respir Crit Care Med.

[4] Mariani F, Gatti B, Rocca A, et al. Pleuroparenchymal fibroelastosis: the prevalence of secondary forms in hematopoietic stem cell and lung transplantation recipients. Diagn Interv Radiol. 2016. 10.5152/dir.2016.15516 PMC5019843 27460284

[5] Beynat-Mouterde C, Beltramo G, Lezmi G (2014). Pleuroparenchymal fibroelastosis as a late complication of chemotherapy agents. Eur Respir J.

[6] Baroke E, Heussel CP, Warth A (2016). Pleuroparenchymal fibroelastosis in association with carcinomas. Respirology.

[7] Huang Z, Li S, Zhu Y (2015). Pleuroparenchymal fibroelastosis associated with aluminosilicate dust: a case report. Int J Clin Exp Pathol.

[8] Reddy TL, Tominaga M, Hansell DM (2012). Pleuroparenchymal fibroelastosis: a spectrum of histopathological and imaging phenotypes. Eur Respir J.

[9] Oda T, Ogura T, Kitamura H (2014). Distinct characteristics of pleuroparenchymal fibroelastosis with usual interstitial pneumonia compared with idiopathic pulmonary fibrosis.. Chest.

[10] Nakatani T, Arai T, Kitaichi M (2015). Pleuroparenchymal fibroelastosis from a consecutive database: a rare disease entity?. Eur Respir J.

[11] Frankel SK, Cool CD, Lynch DA (2004). Idiopathic pleuroparenchymal fibroelastosis: description of a novel clinicopathologic entity. Chest.

[12] Azoulay E, Paugam B, Heymann MF (1999). Familial extensive idiopathic bilateral pleural fibrosis. Eur Respir J.

[13] Cheng SK, Chuah KL (2016). Pleuroparenchymal fibroelastosis of the lung: a review. Arch Pathol Lab Med.

[14] Von der Thusen JH (2013). Pleuroparenchymal fibroelastosis: its pathological characteristics. Curr Respir Med Rev.

[15] Kokosi MA, Nicholson AG, Hansell DM (2016). Rare idiopathic interstitial pneumonias: LIP and PPFE and rare histologic patterns of interstitial pneumonias: AFOP and BPIP. Respirology.

[16] Kusagaya H, Fujisawa T, Enomoto N (2015). Co-occurrence of pneumoperitoneum and pneumothorax in a patient with pleuroparenchymal fibroelastosis. Am J Respir Crit Care Med.

[17] Harada T, Yoshida Y, Kitasato Y (2014). The thoracic cage becomes flattened in the progression of pleuroparenchymal fibroelastosis. Eur Respir Rev.

[18] Watanabe S, Waseda Y, Takato H (2015). Pleuroparenchymal fibroelastosis: distinct pulmonary physiological features in nine patients. Respir Investig.

[19] Sato S, Hanibuchi M, Fukuya A (2014). Idiopathic pleuroparenchymal fibroelastosis is characterized by an elevated serum level of surfactant protein-D, but Not Krebs von den Lungen-6. Lung.

[20] De Lauretis A, Basra H, Hakim W, et al. Pleuroparenchymal Fibroelastosis (PPFE) Predicts Survival in Idiopathic Pulmonary Fibrosis (IPF). 2016:A1142, 1110.1164/ajrccm-conference.2016.1193.1141_MeetingAbstracts.

[21] Rosenbaum JN, Butt YM, Johnson KA (2015). Pleuroparenchymal fibroelastosis: a pattern of chronic lung injury. Hum Pathol.

[22] Yoshida Y, Nagata N, Tsuruta N (2016). Heterogeneous clinical features in patients with pulmonary fibrosis showing histology of pleuroparenchymal fibroelastosis. Respir Investig.

[23] Enomoto N, Kusagaya H, Oyama Y (2014). Quantitative analysis of lung elastic fibers in idiopathic pleuroparenchymal fibroelastosis (IPPFE): comparison of clinical, radiological, and pathological findings with those of idiopathic pulmonary fibrosis (IPF). BMC Pulm Med.

[24] Camus P, von der Thusen J, Hansell DM (2014). Pleuroparenchymal fibroelastosis: one more walk on the wild side of drugs?. Eur Respir J.

[25] Ravaglia C, Bonifazi M, Wells AU (2016). Safety and diagnostic yield of transbronchial lung cryobiopsy in diffuse parenchymal lung diseases: a comparative study versus video-assisted thoracoscopic lung biopsy and a systematic review of the literature. Respiration.

[26] Johannson KA, Marcoux VS, Ronksley PE, et al. Diagnostic yield and complications of transbronchial lung cryobiopsy for interstitial lung disease: a systematic review and meta-analysis. Ann Am Thorac Soc. 2016. 10.1513/AnnalsATS.201606-461SR 27466899

[27] Piciucchi S, Tomassetti S, Casoni G (2011). High resolution CT and histological findings in idiopathic pleuroparenchymal fibroelastosis: features and differential diagnosis. Respir Res.

[28] Sato S, Hanibuchi M, Takahashi M (2016). A patient with idiopathic pleuroparenchymal fibroelastosis showing a sustained pulmonary function due to treatment with pirfenidone. Intern Med.

